# Comparison of Fecal Antimicrobial Resistance Genes in Captive and Wild Asian Elephants

**DOI:** 10.3390/antibiotics12050859

**Published:** 2023-05-06

**Authors:** Kaixun Cao, Yepeng Wang, Xuewei Bai, Jishan Wang, Liting Zhang, Yongjing Tang, Rebecca Caroline Thuku, Wei Hou, Guoxiang Mo, Fei Chen, Lin Jin

**Affiliations:** 1 College of Life Sciences, Nanjing Agricultural University, Nanjing 210095, China; 2021204059@stu.njau.edu.cn (K.C.);; 2 Key Laboratory of Animal Models and Human Disease Mechanisms of Chinese Academy of Sciences/Key Laboratory of Bioactive Peptides of Yunnan Province, Kunming Institute of Zoology, Chinese Academy of Sciences, Kunming 650223, China; 3 College of Veterinary Medicine, Shanxi Agricultural University, Jinzhong 030801, China; 4 Asian Elephant Research Center of National Forestry and Grassland Administration, Kunming 650031, China; 5 Southwest Survey and Planning Institute of National Forestry and Grassland Administration, Kunming 650031, China

**Keywords:** *Elephas maximus*, fecal microbial community, antibiotic resistance genes, wildlife conservation

## Abstract

The Asian elephant (*Elephas maximus*) is a flagship species of tropical rainforests, and it has generated much concern. In this case, the gut bacterial communities of captive and wild Asian elephants are particularly noteworthy. We aim to compare the differences in bacterial diversity and antibiotic resistance gene (ARG) subtypes in fecal samples of Asian elephants from different habitats, which may affect host health. Analyses reveal that differences in the dominant species of gut bacteria between captive and wild Asian elephants may result in significant differences in ARGs. Network analysis of bacterial communities in captive Asian elephants has identified potentially pathogenic species. Many negative correlations in network analysis suggest that different food sources may lead to differences in bacterial communities and ARGs. Results also indicate that the ARG levels in local captive breeding of Asian elephants are close to those of the wild type. However, we found that local captive elephants carry fewer ARG types than their wild counterparts. This study reveals the profile and relationship between bacterial communities and ARGs in different sources of Asian elephant feces, providing primary data for captive breeding and rescuing wild Asian elephants.

## 1. Introduction

The Asian elephant (*Elephas maximus*) is an endangered species according to the International Union for Conservation (IUCN) of Nature’s Red List [1]. As an essential wild Asian elephant habitat, Xishuangbanna concentrates 95% of China’s wild elephants living and breeding in Xishuangbanna National Nature Reserve [2]. With the efforts of the Chinese government and the elephant-human coexistence in southwestern Yunnan and other areas, the Asian elephant population in Xishuangbanna has grown from 101 in 1976 to approximately 184–205 in 2016 [3]. In the past, our studies on wild elephants living in Xishuangbanna focused on the changes in forest area and habitat area [2,4,5]. As the habitat of wild animals changes, human activities are accelerating the spread of antibiotic resistance genes (ARGs) in the wild. They pose a global threat to ecological security and wildlife health. The emergence of antibiotic-resistant pathogens due to the misuse or overuse of antibiotics in human and animal health is a global concern [6]. There are increasing reports of multidrug-resistant Gram-negative infections to antibiotics, such as cephalosporins and carbapenems in fecal microorganisms of domestic animals: pigs, dogs, and horses [7,8,9,10,11]. Previous studies have mainly focused on settings with high human antibiotic use, such as hospitals and intensive farms [8,9,10]. However, the widespread use of antibiotics has resulted in trace amounts in most environments, including those mentioned above [12,13]. Chronic exposure to a mixture of hundreds of residual antibiotics, even at low concentrations, is likely to increase the abundance of antibiotic resistance genes (ARGs) via mobile genetic elements (MGEs) [14,15,16,17]. With the widespread use of birdshot sequencing technology for environmental microbial monitoring and various fields, increasingly comprehensive studies on the composition of wildlife gut bacteria and their associated ARG are being conducted [18]. ARG spread into the environment threatens human health [11] and harms wildlife’s health [19]. However, little research has been reported on ARG in Asian elephants. Monitoring wild elephant feces not only provides an understanding of the gut bacterial status of wild elephants but can also identify potential melting pots of novel ARG combinations that are more harmful to humans [20].

As mentioned above, in the past years, wild animals have been recognized as vectors and secondary sources of ARB for humans and animals [21,22]. Although wild animals are not directly exposed to antibiotics, they are affected by their extensive use in human and veterinary medicine [8,12]. Compared with wild animals, captive wild animals have more frequent contact with humans, which may account for abundance of ARGs carried by gut microbes, potentially causing health damage to the host [23,24]. This is because the microbiota imbalance caused by ARGs can negatively affect the host’s health in several ways and over a long period [24]. In addition, captivity and fixed diets may reduce the diversity of wildlife gut microbes [25,26]. The host gut microbial diversity shows a tendency to decrease in many disease states [27]. This study’s results may help improve the captive conditions and the health status of wild animals and optimize their dietary composition. Therefore, to identify factors that may enhance the health status of Asian elephants, it is important to characterize and compare the gut microbial communities and ARG profiles of captive and wild Asian elephants. A study by Li et al. on the comparative and functional analysis of the fecal microbiome of semi-captive Asian elephants of different ages revealed extensive ARG carriage in semi-captive Asian elephants [28]. However, differences in intestinal bacterial communities and ARG between captive and wild Asian elephants have not been reported.

This study used metagenomic sequencing to profile the fecal bacterial communities and resistance groups of captive and local wild Asian elephants in Xishuangbanna, where wild Asian elephants were at the start of their northern migration in 2021. The results of this study will help to understand the effects of different living environments on the gut bacterial communities and ARGs of Asian elephants and provide baseline data for developing more detailed conservation strategies for Asian elephants.

## 2. Results

### 2.1. Fecal Metabolomics Profile

**Figure 1:**
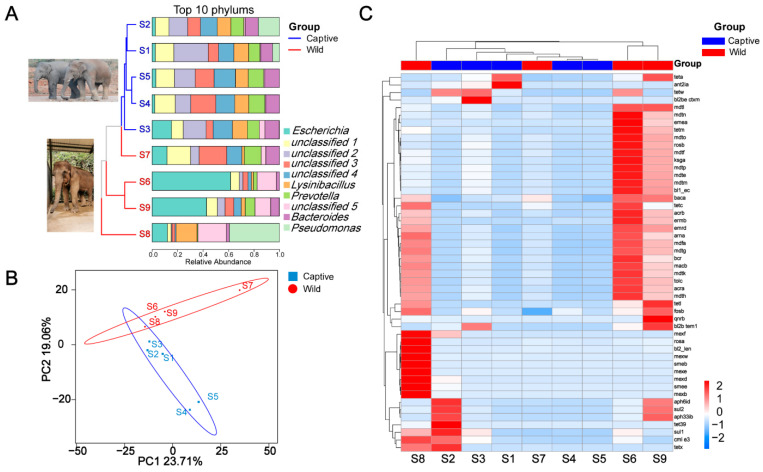
(**A**) Stacked histograms of the top ten clade levels of bacterial abundance in captive and wild Asian elephants, with a developmental tree based on OTUs on the left; (**B**) PCoA based on ARGs carried by captive and wild Asian elephant gut bacteria; (**C**) Heat map of the absolute abundance of bacterial ARGs genes in the feces of each Asian elephant species.

From the Supplemental Table S1, we identified 138 ARGs from nine elephant fecal samples. Only 24 ARGs were present in all elephant feces, representing 17.39% of all ARGs. Genes *bacA* and *acrB* were the most common, followed by *tetL*, but the distribution of the gene *tetL* in wild and captive elephant fecal samples was significantly different. The gene *tetL* was present in 80% of wild elephant fecal samples and reads of this genus were almost negligible in S1, S2, and S3. Based on Figure 1A, the captive and wild Asian elephants show apparent fecal bacteria at the top ten phylum level. However, sample S3 appears to be an exception, with a closer distribution of bacterial abundance at the top ten phylum level in the long fecal samples of captive Asian elephants and more significant variation in each sample of wild Asian elephants. *Unclassified4* and *Lysinibacillus*, which were more evenly distributed in the captive elephant fecal samples, were lower in the wild Asian elephant fecal samples. We used the Hill number to analyze the beta diversity of antibiotic resistance classes annotated by CARD. The results show that beta indices show significant variability when q ≥ 2 (the Supplemental Figure S1; *p* < 0.01), indicating that antibiotic resistance classes with high abundance differ significantly more among wild than domesticated elephants. PCoA of ARGs profiles showed that ARGs of bacteria in wild and captive elephant fecal samples, except S3 and S7, were clustered and separated from each other in the first dimension, representing 42.77% of total variation (PERMANOVA test, *p <* 0.01; Figure 1B). Similarly, heat map analysis revealed that the ARGs in wild elephant fecal samples other than S7 were clustered and distinct from those of captive elephants. In addition, the abundance of ARGs to aminoglycosides, polypeptides, tetracyclines, macrolides, and polypharmacy was increased in the elephant fecal samples group.

### 2.2. Linear Discriminant Analysis Effect Size (LEfSe) Analysis

**Figure 2:**
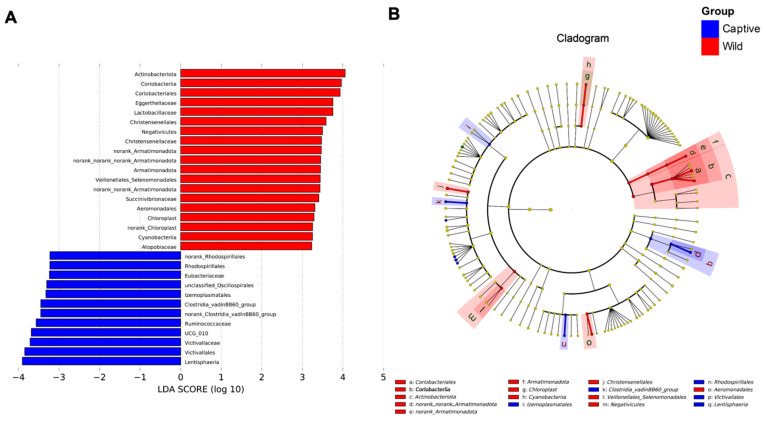
Linear discriminant analysis effect size (LEfSe) analysis results showed significant differences between the fecal bacterial communities of captive and wild Asian elephants. (**A**) Log10-based LDA score of specific bacteria enrichment in two different lifestyles of the Asian elephant (captive and wild). (**B**) Cladogram showing the most differentially abundant taxa identified by LEfSe. Red indicates clades enriched in the wild elephant, whereas blue indicates clades increased in the captive elephant.

The LEfSe analysis was performed to describe the specific bacterial groups in the fecal microbiota of captive versus wild Asian elephants, as shown in Figure 2. The LDA log10-based score highlighted the abundance of plotted serpentine rock and fecal samples from captive and wild Asian elephants, as shown in Figure 2A. The results showed that the abundance of *Ruminococcaceae*, *Ruminococcaceae* UCG-010, *Victivallaceae*, *Victivallales,* and *Lentisphaeria* was significantly enriched in captive Asian elephant feces samples, whereas *Actinobacteriota*, *Coriobacteriia*, *Coriobacteriales*, *Eggerthellaceae,* and *Lactobacillaceae* were increased considerably in wild Asian elephants’ feces samples. The cladogram was also plotted to show their differences in taxonomical hierarchies (Figure 2B).

### 2.3. Microbiome-Metabolome Associations

**Figure 3:**
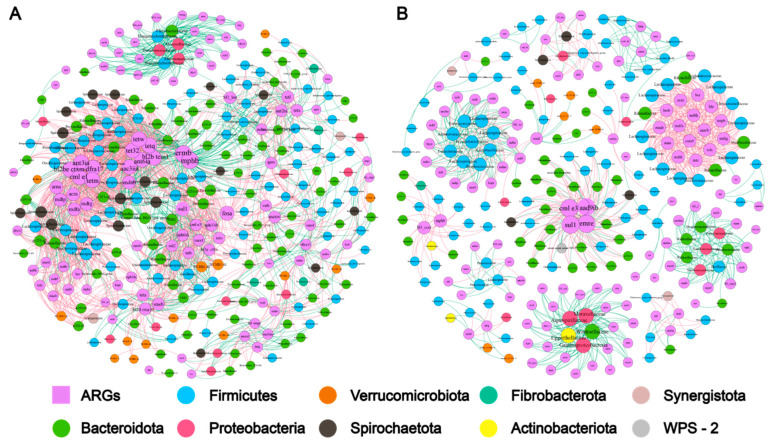
Association network of gut bacteria and ARGs by captive and wild Asian elephant modules. Only correlations between statistically significant (*p* < 0.01) and strong (ρ ≥ 0.6) are shown. Red solid lines indicate negative correlations and green indicates positive correlations. Different colors indicate different microbial gates, and the number on each node means the number of OTUs clustered at 97% similarity. The circles consist of many node representation modules. (**A**,**B**) represent the gut bacterial network from captive and wild Asian elephants.

As shown in the Supplemental Figure S2, there was a significant Spearman’s rank correlation (Spearman’s ρ = 0.62, *p*-value < 0.1) between the bacteria diversity and the ARG diversity. The co-occurrence patterns among ARG subtypes were explored using network inference based on strong (ρ > 0.6) and significant (*p*-value < 0.01) correlations. The co-occurrence analysis of captive elephant fecal bacteria resulted in 319 nodes (including 216 bacterial species and 103 ARG subtypes) and 2052 edges (764 positive and 1288 negative correlations), and a modularity index of 0.596 for the co-occurrence analysis of captive elephant fecal bacteria, indicating a modular network structure (Figure 3A). The co-occurrence analysis of wild elephant fecal bacteria resulted in only 269 nodes (including 115 bacterial species and 114 ARG subtypes) and 936 edges (449 positive and 487 negative correlations), far fewer than for captive elephants. The modularity index for wild elephant fecal bacteria was 0.895, indicating that the network has a higher degree of modularity than for the captive type. There were 318 nodes (including 225 bacterial species and 137 ARG subtypes) and 1239 edges (365 positive and 874 negative correlations), with a modularity index of 0.585, which suggested that the network had a modular structure (Figure 3B). It is clear that the patterns associated with the gut bacteria of captive Asian elephants are more complex than those of wild Asian elephants. More OTUs were significantly associated with ARGs in captive elephants than in wild elephants, but they belonged to only seven phyla: *Bacteroidota*, *Firmicutes*, *Spirochaetota*, *Verrucomicrobiota*, *Proteobacteria*, *Fibrobacterota*, and *Synergistota*; however, there are more subtypes of ARGs among wild elephants, and although the number of OTUs is smaller than that of captive elephants, they belong to nine phyla: *Firmicutes*, *Bacteroidota*, *Proteobacteria*, *Verrucomicrobiota*, *Spirochaetota*, *Actinobacteriota*, *Synergistota*. *Actinobacteriota*, *Synergistota*, *Fibrobacterota,* and *WPS-2*.

### 2.4. Overview of Resistance Gene Abundance

**Figure 4:**
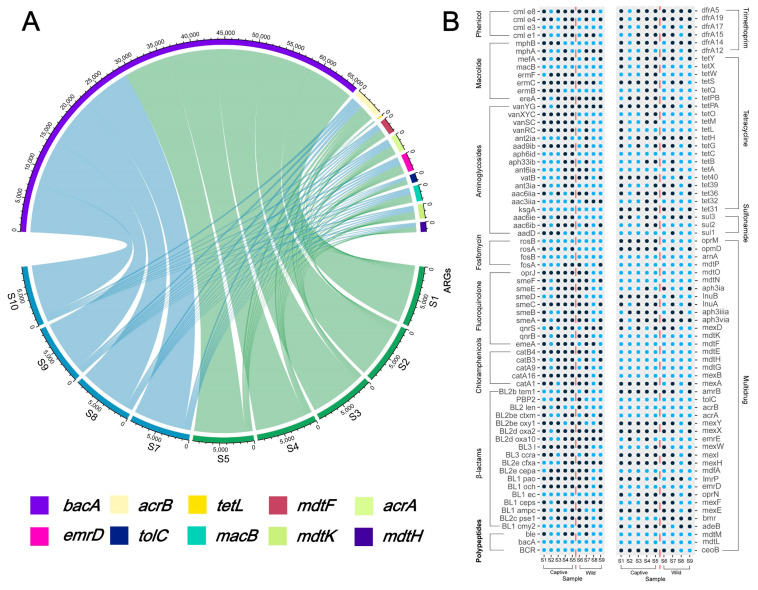
(**A**) Circos of each Asian elephant species and the top ten abundance ARGs; (**B**) Relationship between the subtypes of ARGs each Asian elephant possesses and the corresponding antibiotic type, with blue representing the presence of that ARGs subtype in that sample and black indicating its absence.

As shown in Figure 4A, the number of ARGs associated with multiple drug resistance was the highest in both wild and captive Asian elephant gut bacteria at 34.23% and 26.13%, respectively, followed by tetracycline resistance genes (17.12% and 14.41%), with higher aminoglycosides antibiotic resistance genes in wild Asian elephant gut bacteria (14.41% and 11.71%), and captive Asian elephant gut bacteria carrying more β-lactam resistance genes were higher in wild Asian elephants (14.41% and 11.71%). In comparison, captive Asian elephants had more β-lactam resistance genes (12.61% and 13.51%). The number of ARGs is shown in the Circos. We selected the top ten ARG subtypes in terms of abundance for Circos analysis and found that *bacA* was the most contributing ARG subtype with respect to ARG abundance in both wild and captive elephant gut bacteria. The results show an extremely high proportion of *bacA*, the dominant persistent ARG in drinking water worldwide, which is regarded as the intrinsic gene of bacteria. We also looked for evidence through a Procrustes analysis, as the statistical efficacy of Procrustes also proved superior to that of the Mantel test. Unlike the other results, Proust analysis data are shown in the Supplemental Figure S3; a better agreement was not achieved for samples S1–S9 in the Procrustes analysis, suggesting that the potential relationship between ARGs and bacteria from different fecal samples is poor. Nevertheless, some of the same factors may make the ARGs converge in elephant fecal samples from different environments. Figure 4B demonstrates the presence or absence of the ARGs gene in different samples from S1–S9 through a scatter plot (blue indicates the presence of the gene in that sample, and black indicates the opposite). The results showed that the carriage rates of ARGs in captive and wild Asian elephants were 71.74% and 82.61%, respectively, and that the fecal bacteria of wild Asian elephants carried more subtypes of ARGs compared with those of captive Asian elephants.

## 3. Discussion

In this study, we collected fresh fecal samples from five captive and four wild Asian elephants and used metagenomic sequencing to characterize their microbiome and antibiotic resistance gene (ARG) profiles. The four wild Asian elephants were traced and named during the well-known migration in 2021 overcoming the uncertainty of the defecation habits of wild animals and the potential health risks to humans. The results showed that (i) the fecal microbiome composition differed significantly between captive and wild elephants, as evidenced by differences in beta diversity (Hill numbers) and clustering analysis; (ii) annotation of UniGenes based on KEGG, eggNOG, and CAZyme revealed similar differences in antibiotic-resistant groups in the fecal samples of captive and wild elephants; and (iii) network analysis revealed significant differences in bacterial communities associated with ARGs. This study on the bacterial communities and ARGs of captive and wild Asian elephants reflects the comprehensive resistance profiles of these animals under different living conditions. It reveals potential health risks for both captive and wild Asian elephants.

The PCoA, heat map, LEfSe, and beta diversity results based on relative abundance showed significant differences between the Asian elephant fecal bacterial community and ARGs in natural and captive environments. The study revealed that fecal bacteria from wild Asian elephants carry more ARGs. Indeed, the spread of antimicrobial resistance in wildlife has become a hot topic recently [19,29]. It is generally accepted that the number and variety of ARGs carried by animals increase as they become exposed to human activities [30,31,32,33]. However, we found that both the number and subtypes of ARGs were higher in the wild than in captive Asian elephant fecal samples, which is inconsistent with the results of the above study. The spread of antibiotic resistance has increased with the growing use of antibiotics and the increasing impact of human activities on the natural environment [34]. Wild Asian elephants have more space and a more comprehensive range of food sources than those living in captivity. Antibiotics are naturally produced by environmental bacteria and fungi. For mammals, food source and food type are usually the main prototypes that shape the type of gut microbiota [35]. Therefore, ARGs detected in the fecal samples of the wild Asian elephants may be caused by the widely dispersed food source. In addition, the taste differences may explain the presence of anomalous individuals. Our results based on β diversity (Hill number) showed decreasing sensitivity of the index to rare species (increasing the order q), and the β diversity of bacterial communities in Asian elephant feces showed significant differences between the two environments. Our results were consistent with those of Keylie et al. [36]. The β diversity of wildlife microbial communities is often higher than that of captive wildlife. The provision of similar diets in captivity may have exerted selective pressure on the abundance of bacterial communities in the gut of Asian elephants [37] in addition to the fact that keeping wild animals in captivity reduces the impact of environmental change [38].

LEfSe analysis identified 31 characteristic taxa related to wild and captive groups. Bacteria related to cellulose, hemicellulose, and lignin degradation, such as *Ruminococcaceae*, *Coriobacteriales*, *Actinobacteriota*, etc. [39,40,41,42] were highly abundant in captive Asian elephants and wild Asian elephants. However, there were significant differences in bacterial communities affecting health (including positive and negative effects) between captive and wild elephants. Several bacterial communities, *Lentisphaeria* and *Clostridiales* vadinBB60 group, are highly abundant in the fecal samples of captive elephants. Although *Lentisphaeria* may be a significant lignocellulosic degrader in the rumen (Gharechahi et al. 2021), *Lentisphaeria* is possibly associated with acute stroke (2022). A report by Ning et al. also suggests that *Lentisphaeria* may also be associated with amyotrophic lateral sclerosis [43]. The *Clostridiales* vadinBB60 group also poses a threat to the health of the host. The increase in the *Clostridiales* vadinBB60 group may be responsible for the reduced neuroplasticity of the central nervous system (CNS) [44], and the effects of the *Clostridiales* vadinBB60 group on the CNS are also reflected in the aging process [45]. Although the hosts in the above studies were all humans and there have been no reports of the effects in elephants, this is a cause for worry given the scarcity of Asian elephant populations. Compared with the results of LEfSe on the bacterial communities of Asian elephants in captivity, wild Asian elephants fare much better. Animals closer to their natural environment have higher levels of *Christensenellales*, and broiler health studies have confirmed that high levels of *Christensenellales* benefit host health [46,47]. These differences, and the potential health benefits of these bacterial communities, may help to improve the health of Asian elephants in rescue, treatment, and captivity in the future, and may help to keep them in a healthier state in zoos and sanctuaries.

Past studies have suggested that ARGs change significantly with changes in microbial communities [48,49], as evidenced by Spearman’s rank correlation of ARGs with OTU numbers in our results. We need to obtain a more specific symbiotic relationship between ARGs and bacterial communities through network analysis. The results of network analysis based on strong (ρ > 0.6) and significant (*p*-value < 0.01) correlations show that ARGs in captive Asian elephant fecal samples have more potential ARG hosts. However, wild elephants have a more significant number of ARG subtypes. The *Rikenellaceae* RC9 gut group was the most abundant in both captive and wild Asian elephants in the network analysis. The wild Asian elephant *Rikenellaceae* RC9 gut group still showed a strong negative correlation with most ARGs (green line in Figure 3B), while the *Rikenellaceae* RC9 gut group was mainly associated with crude fiber in the diet [50]. We speculate that the crude fiber component of the captive elephant diet might be the main reason for carrying so many ARG subtypes. Other highly abundant bacterial communities, such as unclassified *Lachnospiraceae* and unclassified p-251-o5, are common microorganisms in the ruminant gut [51,52,53]. The unclassified *Lachnospiraceae* are a group of bacteria that play an essential role in cellulose digestion [54]. Both are highly correlated (positively or negatively) with various ARGs. The abundance of unclassified p-251-o5 was higher in captive Asian elephants than in wild Asian elephants, while the abundance of unclassified *Lachnospiraceae* was slightly lower. Previous studies have suggested that unclassified *Lachnospiraceae* are more abundant in the rumen of healthy animals and less abundant in p-251-o5 [53]. Notably, in captive Asian elephants, *Treponema* was second only to the highest gut group *Rikenellaceae* RC9 in abundant bacterial communities significantly associated with ARG. In contrast, in wild Asian elephants, *Treponema* was only tenth in abundance. *Treponema* is a joint group of pathogenic bacteria and a carrier of ARG [55,56]. These differences in bacterial abundance results confirm that captive Asian elephants have unfavorable health conditions compared with wild Asian elephants. The network analysis results revealed a higher number of negative correlations between OTUs and ARGs in the gut bacteria of captive Asian elephants than in wild ones. This suggests a higher degree of ecological niche overlap among the gut bacteria of captive Asian elephants, which may result from more intense competition for food resources [57]. Additionally, the higher ecotopic overlap of captive Asian elephant gut bacteria and the higher abundance of bacteria associated with fiber/semi-fiber degradation indicate that reduced food abundance in captive Asian elephants could be the primary cause of the differences in bacterial communities and ARGs observed between captive and wild populations. These findings highlight the importance of maintaining a diverse and abundant food supply for captive Asian elephants, as it could help to restore their gut microbiome diversity and reduce the potential for ARG contamination and transmission.

The following top 10 abundances of ARGs were edetected in captive and wild Asian elephant fecal samples: *bacA*, *acrB*, *tetL*, *mdtF*, *acrA*, *emrD*, *tolC*, *macB*, *mdtK*, *mdtH*, accounting for 51.47% of the total ARGs detected and mainly associated with multidrug, polypeptide, and quinolone resistance. The numbers and ratios of ARG subtypes found in the Circos are similar to the results from the Antarctic soils and along the Yarlung Tsangpo River [58,59], with *bacA* generally observed in the most significant proportion of samples from relatively pristine environments [60] because the *bacA* gene is typically considered to be intrinsic to bacteria [49]. This result suggests that despite the challenges faced by captive and wild elephants in Xishuangbanna (as mentioned in our results above), they still live in a relatively pristine environment, which is an indicator of the conservation efforts for Asian elephants in southwest Yunnan. The *acrA* and *acrB* genes are common multidrug resistance genes in many Gram-negative bacteria and act primarily by encoding multidrug efflux pumps [61,62]. In contrast, *tetL* is primarily associated with tetracycline resistance [63] by reducing intracellular tetracycline accumulation through efflux. In addition to the multidrug resistance efflux genes mentioned above, *emrD*, *mdtF*, *mdtK*, *mdtH*, *macB,* and *TolC*, with *macB* and *mdtK* require *TolC* to function [64,65]. The plot of each Asian elephant sample and whether they carried ARG shows that the wild Asian elephant carries more ARG. In recent years, the number of reports of wildlife carrying ARG subtypes has increased [19,66,67], which raises additional concerns about the potential role of wildlife in the spread of ARG, as well as the carrying of ARG subtypes that pose a threat to humans, as well as the virulence profile and public health issues that may occur as a result [67]. In addition, Asian elephants are often essential for local seed dispersal [68]. Therefore, consideration of the health of Asian elephants in Xishuangbanna is also crucial for local ecological health.

## 4. Conclusions

This study identified differences in gut bacterial communities and ARGs between captive and wild Asian elephants. We found that captivity significantly reduced the diversity of most abundant bacterial communities in the gut of Asian elephants and identified two potentially pathogenic bacterial groups (p-251-o5 and Treponema) in captive Asian elephants. These results provide important baseline data for understanding the intestinal flora of Asian elephants and the relationship between antibiotic resistance and health. Maintaining and restoring the gut microbiome diversity and metabolic potential of captive Asian elephants through a richer food variety may help improve the health status of Asian elephants. We suggest that reducing ARG contamination and transmission while increasing the protection of plants that local Asian elephants feed on might facilitate the long-term conservation of Asian elephants and the maintenance of a robust ecosystem.

## 5. Materials and Methods

### 5.1. Sample Collection and Pretreatment

We collected and analyzed feces from five captive and four wild elephants at the northward migration’s departure point in 2021. S1–S5 are fecal samples isolated from captive elephants fed without artificially added antibiotics, while S6–S9 are fresh feces samples from the north-migrating elephant herd collected in Mojiang on 24–25 March 2021. Detailed information about animals is shown in Table S1. Fecal samples were packed into sterile bags immediately after these animals’ defecation, frozen in dry ice, and stored at −80 °C until DNA extraction. There was no harm or intervention to the animals; therefore, an ethical review process was not required per the institutional guidelines.

### 5.2. DNA Extraction and PCR Amplification

Following the manufacturer’s instructions, total bacterial genomic DNA was extracted from elephant fecal samples using the NEXTFLEX™ Rapid DNA-Seq Kit (Bioo Scientific, Austin, TX, USA). Index codes were added to ensure the correct sequence was assigned to the proper sample. Each DNA sample was fragmented to 400 bp using Covaris M220 (Covaris, Woburn, MA, USA) and then screened with magnetic beads to remove self-linked fragments. PCR amplification was then performed using ransGen AP221-02: TransStart Fastpfu DNA Polymerase (Beijing, China, TransGen Biotech) and AxyPrep DNA Gel Extraction Kit (Axygen Biosciences, Union City, CA, USA) to extract PCR products from 2% agarose gels and purified for quantification using a Quantus™ Fluorometer (Promega, Madison, WI, USA). Sequencing was then performed on Illumina’s Miseq PE300/NovaSeq PE250 platform.

### 5.3. Bioinformatics and Statistical Analysis

After the sequencing, the raw bacterial sequences were processed using the QIIME pipeline [69]. Sequences with a quality score of less than 25 and a length of less than 200 bp were removed, and the remaining sequences were assigned to fecal samples based on their unique barcodes. The barcodes and primers were removed before merging all the qualified sequences, and representative sequences were obtained after removing redundancies. Then the optimized sequences were clustered into operational taxonomic units (OTUs) using UPARSE 7.1 with a 97% sequence similarity level [70,71]. The most abundant sequence for each OTU was selected as a representative sample.

Statistical analysis and mapping included linear discriminant analysis (LDA) effect sizes (LEfSe) using LEfSe software (default LDA score of 3) to determine differences in microbial taxa between the two groups. Annotation of resistance genes was as follows: UniGenes were compared with the Comprehensive Antibiotic Resistance Database (CARD; https://card.mcmaster.ca/; accessed on 5 January 2022.) using Resistance Gene Identifier (RGI) software. The relative abundance of antibiotic resistance ontology terms (AROs) was determined based on the comparison results.

Bioinformatics analysis of the fecal microbiota was conducted using the Majorbio Cloud platform (https://cloud.majorbio.com; accessed on 5 January 2022). Based on the OTUs information, ‘MetagenomeDiversity.R’ provided by Ma was used to calculate Hill-number-based alpha and beta diversity indices [72]. Heat map construction, non-metric multidimensional scaling (NMDS), principal component analysis (PCA), principal coordinate analysis (PCoA), and network analysis were also performed in R (http://www.r-project.org/; accessed on 5 January 2022) to visually compare overall differences in microbial taxonomic composition, functional genes, and antibiotic resistance.

## Data Availability

All data are staged at https://cloud.majorbio.com/ and are available on request from the corresponding author. The data are not publicly available due to privacy.

## Supplementary Materials

The following supporting information can be downloaded at: https://www.mdpi.com/article/10.3390/antibiotics12050859/s1, Figure S1: beta diversity of ARGs; Figure S2: Spearman’s rank correlation between the number of OTUs and the number of ARGs; Figure S3: Procrustes analysis shows that captive and wild Asian elephants have similar clustering patterns in their fecal bacterial profiles and ARGs gene content; Table S1: ARGs list.
