# Supplementary material for: Comparison of Fecal Antimicrobial Resistance Genes in Captive and Wild Asian Elephants
Source: Antibiotics (Basel). 2023 May 6;12(5):859. doi: 10.3390/antibiotics12050859 (PMC10215966; doi:10.3390/antibiotics12050859)
Supplement: Supplementary file 1 [file antibiotics-12-00859-s001.zip › Supplementary Information.docx]

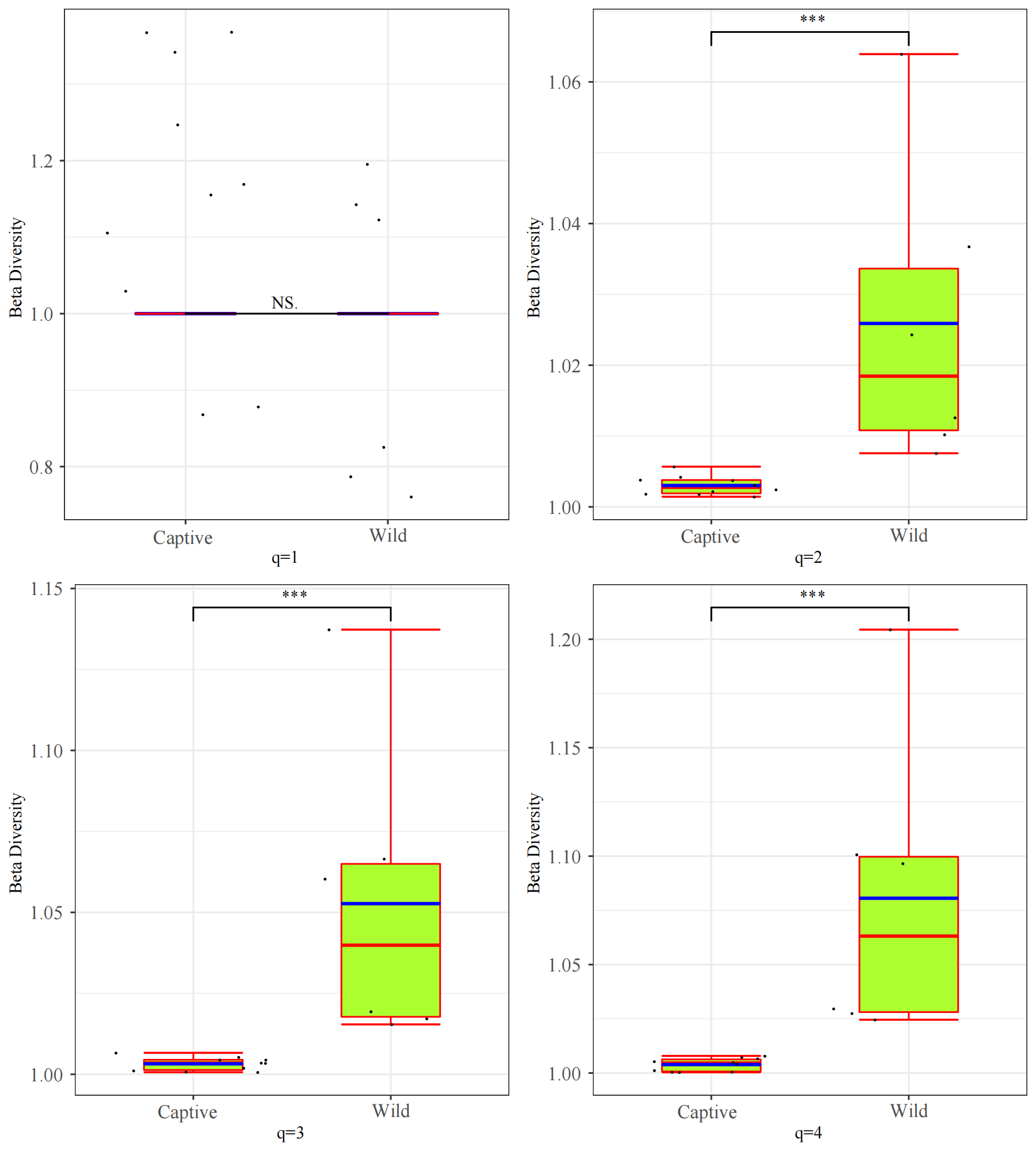


**Supplemental Figure 1.** beta diversity of ARGs (q = 0 describes the number of species, q > 0 describes species with high abundance). ^***^P<0.001.


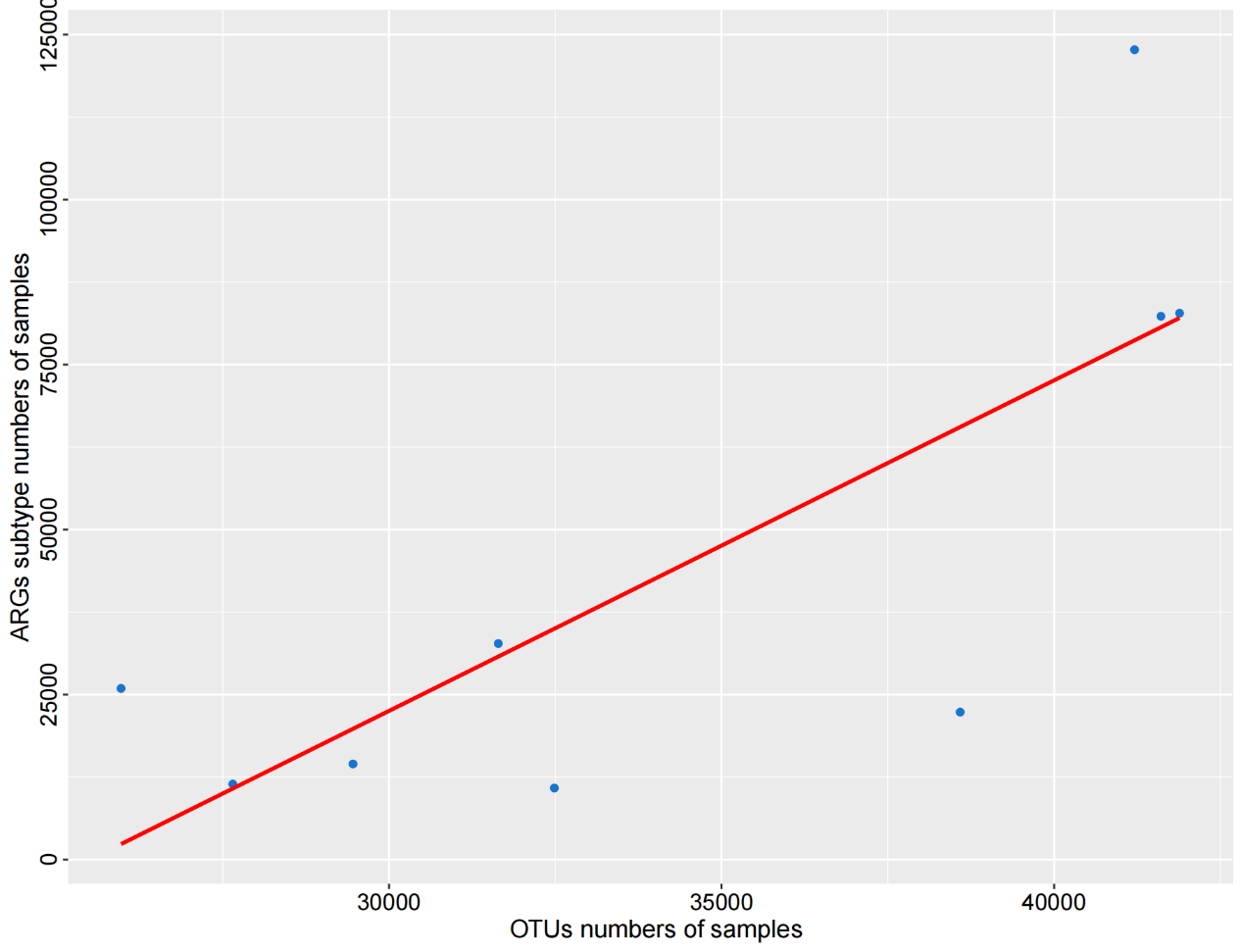


**Supplementary Figure 2.** Spearman's rank correlation between the number of OTUs and the number of ARGs.


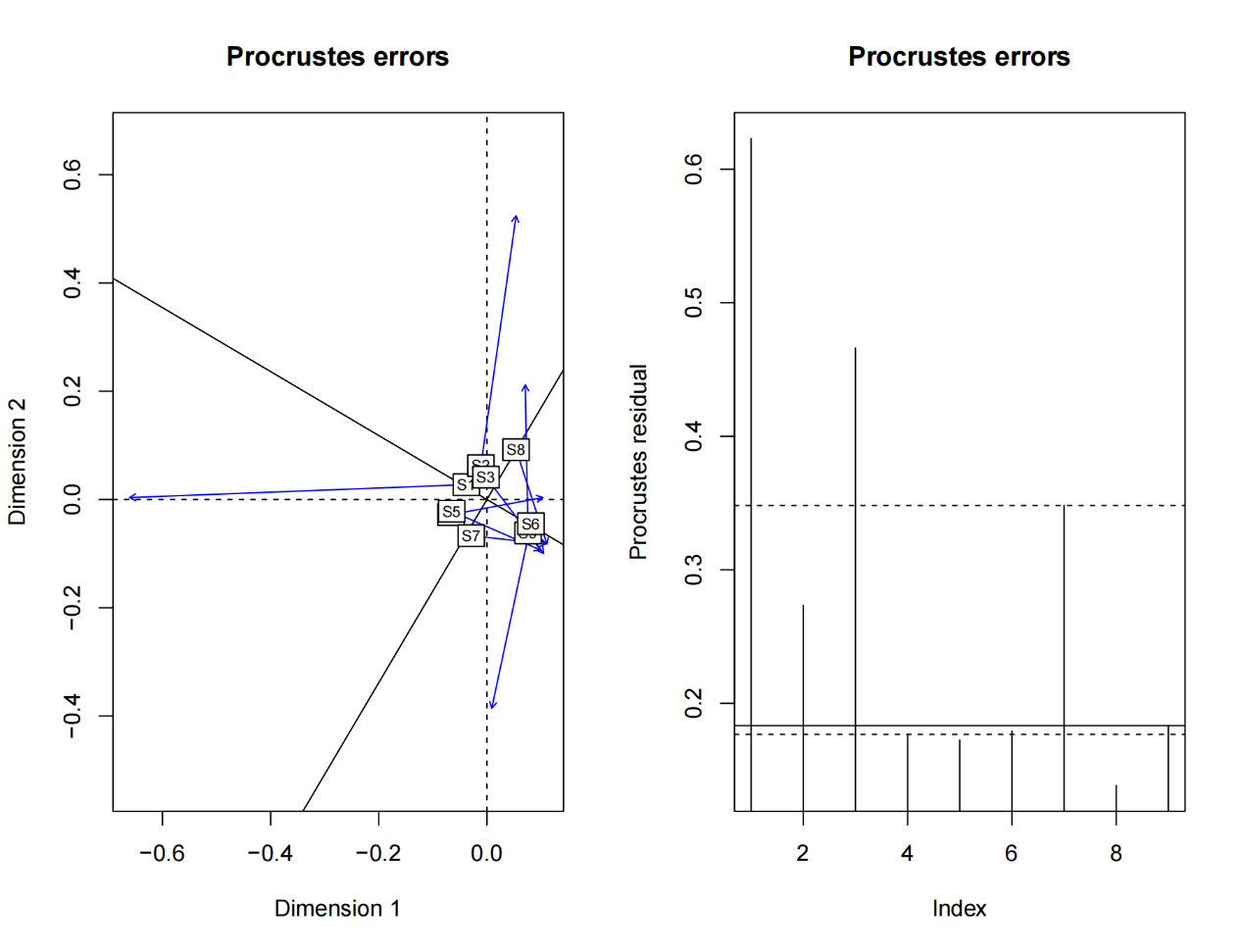


**Supplementary Figure 3.** Procrustes analysis shows that captive and wild Asian elephants have similar clustering patterns in their faecal bacterial profiles and ARGs gene content.
